# The Developmental Trajectory of Self-Esteem Across the Life Span in Japan: Age Differences in Scores on the Rosenberg Self-Esteem Scale From Adolescence to Old Age

**DOI:** 10.3389/fpubh.2020.00132

**Published:** 2020-08-06

**Authors:** Yuji Ogihara, Takashi Kusumi

**Affiliations:** ^1^Division of Cognitive Psychology in Education, Graduate School of Education, Kyoto University, Kyoto, Japan; ^2^Faculty of Science Division II, Tokyo University of Science, Tokyo, Japan

**Keywords:** self-esteem, age difference, developmental trajectory, culture, self-competence, self-liking, Japan, Rosenberg self-esteem scale

## Abstract

We examined age differences in global self-esteem in Japan from adolescents aged 16 to the elderly aged 88. Previous research has shown that levels of self-liking (one component of self-esteem) are high for elementary school students, low among middle and high school students, but then continues to become higher among adults by the 60s. However, it did not measure both aspects of self-esteem (self-competence and self-liking) or examine the elderly over the age of 70. To fully understand the developmental trajectory of self-esteem in Japan, we analyzed six independent cross-sectional surveys. These surveys administered the Rosenberg Self-Esteem Scale, which measured both self-competence and self-liking, on a large and diverse sample (*N* = 6,113) that included the elderly in the 70s and 80s. Results indicated that, consistent with previous research, for both self-competence and self-liking, the average level of self-esteem was low in adolescence, but continued to become higher from adulthood to old age. However, a drop of self-esteem was not found over the age of 50, which was inconsistent with prior research in European American cultures. Our research demonstrated that the developmental trajectory of self-esteem may differ across cultures.

## Introduction

Self-esteem, which is the positivity of a person's global evaluations of the self [e.g., Baumeister et al. (1)], is one of the most famous indicators of mental health. To maintain good mental health, it is important to have a positive view of the self to some extent.

The average level of self-esteem changes across the life span along with changes in one's capacities (e.g., social, cognitive) and surrounding environments (e.g., social, economic). Uncovering the developmental trajectory of self-esteem across the life span is important at least for two reasons. First, it is crucial to reveal the effects of basic demographic variable on self-esteem. Age is one of the most frequently examined demographic variables. Thus, how age influences self-esteem should be investigated. Furthermore, when researchers are interested in the effects of other variables (e.g., socio-economic status, interpersonal relationships) on self-esteem, they should control for basic demographic variables that might confound these effects (e.g., age, gender). To statistically control for the effect of age, it is imperative to know in advance how age is associated with self-esteem. Second, investigating the developmental pattern across the lifetime contributes to understanding how self-esteem is formed, maintained, and influenced by changes in one's capacities and surrounding environments. For instance, finding two periods when self-esteem declines can estimate that a consistent factor common to the two periods might decrease self-esteem.

Revealing the developmental trajectory of self-esteem is also important for public health at least for two reasons. Such knowledge contributes to promoting public mental health by providing empirical evidence about the developmental pattern of self-esteem. First, knowing when self-evaluation tends to become negative over the life span facilitate effective prevention and provision. For example, parents and teachers can pay more attention to and provide more resource to people in the periods at higher risk. Second, knowing the period when self-evaluation is at high risk for turning negative can facilitate effective interventions and responses. Interventions and responses that are necessary depend on age categories (e.g., adolescence, old age). Moreover, people that are in the periods of lower self-esteem might feel relieved if they understand that they are not special and it is rather natural to have relatively low self-esteem during specific developmental stages.

Previous research especially in European American cultures has provided empirical evidence about the developmental trajectory of self-esteem over the life course. However, is this developmental pattern of self-esteem consistent across cultures?

### Age Differences in Self-Esteem in European American Cultures

A large amount of research has investigated the developmental trajectory of self-esteem in European American cultures (especially in the U.S.; for reviews, see Orth and Robins (2); Robins and Trzensniewski (3). Robins et al. (4) investigated age differences in self-esteem from a broad range of population aged 9 to 90 years old in the U.S. They found that self-esteem is high in childhood, low in adolescence, but then continues to become higher in adulthood. Then, self-esteem peaks around the mid-60s, and shows a drop afterward. Moreover, Orth et al. (5) explored the developmental trajectory of self-esteem from young adults aged 25 to the elderly aged 104 by analyzing longitudinal data in the U.S. They showed that self-esteem increases from young adulthood through middle age, but then decreases from around the age of 60. In addition, Orth et al. (6) investigated the life-span development of self-esteem from adolescents aged 16 to the elderly aged 97 by examining other longitudinal data in the U.S. They demonstrated that self-esteem rises from adolescence to middle adulthood, peaks at about age 50, and declines in old age. This pattern of developmental change has been found not only in the U.S., but also in Germany. Orth et al. (7) examined the development of self-esteem from adolescents aged 14 to the elderly aged 89 by analyzing a longitudinal study. Results indicated that self-esteem increases from adolescence to middle adulthood, reaches a peak at about age 60 years, and decreases in old age in Germany.

Studies have shown that self-esteem reaches a peak in one's 50s or 60s, and then sharply drops in old age (4–7). This is a characteristic change, so it is important to reveal about when self-esteem peaks across the life span. This drop is thought to occur mainly for two reasons [e.g., Robins et al. (4); Robins and Tresniewski (3)]. The first is the loss of things that are important to one's evaluation of oneself. These include the loss of socioeconomic positions or roles due to retirement, loss of close others (e.g., spouse, romantic partner), and a reduction in one's abilities (e.g., physical, cognitive). The second is a change in attitudes toward oneself. The elderly come to accept their limitations and faults, leading them to have more humble, modest, and balanced perspectives toward themselves.

### Age Differences in Self-Esteem in Japan

Previous research has shown that self-esteem is profoundly affected by culture [e.g., Heine et al. (8); Schmitt and Allik (9)], leading to the possibility that the developmental trajectory of self-esteem may differ across cultures. Thus, it is important to investigate the developmental change in self-esteem in cultures other than America and Europe^1^.

Prior research examined age differences in self-esteem from elementary school students aged 10 to the elderly in their 60s by analyzing cross-sectional data from a large, representative and diverse sample in Japan (12). It showed that levels of self-esteem were high for elementary school students, low among middle and high school students, but then gradually continued to become higher among adults, consistent with the pattern obtained in European American cultures (2, 3). Moreover, previous research has indicated the same pattern of age differences in self-esteem from middle school students to the elderly in their 60s by analyzing another independent and large-sample survey (13).

However, previous research had two limitations. First, it did not directly examine age differences in global self-esteem in Japan. Prior research investigated age differences in self-esteem by focusing on one component of self-esteem: self-liking [“our affective judgment of ourselves, our approval or disapproval of ourselves, in line with internalized social values” ((14), p. 325); also see, Tafarodi and Milne (15); Tafarodi and Swann (16)]. It has been shown that self-esteem consists of self-liking and self-competence (“the overall sense of oneself as capable, effective, and in control”; (14), p. 325), which are strongly correlated with each other and construct self-esteem. Thus, it is strongly predicted that age differences in self-esteem would be consistent with those in self-liking. However, this has not been examined empirically. Although we do not have strong evidence, it is possible that patterns of age differences in self-competence are different from patterns of age differences in self-liking. To reveal the developmental trajectory of global self-esteem, it is desirable to directly investigate age differences in self-esteem by capturing both of its aspects simultaneously.

Second, previous research did not sufficiently investigate age differences in self-esteem in the elderly over the age of 70, leaving the developmental trajectory of self-esteem after the age of 70 in Japan unclear. Previous research in European American cultures has indicated that the average level of self-esteem drops sharply in the elderly period (2, 3). To capture the whole picture of the developmental trajectory of self-esteem in Japan, it is necessary to investigate whether this sharp drop is also found among the elderly in Japan. Many studies have shown that people in Japan have more humble, modest and balanced attitudes toward themselves compared to people in European American cultures [e.g., Heine et al. (8); Heine and Hamamura (17)]. Given that the sharp decline in self-esteem observed in European American cultures may be caused by increases in such attitudes in old age, it is possible that a decline may be absent or less sharp in Japanese older adults. Indeed, one prior study did not find a drop in self-liking between the ages of 50 and 69, which implies that a decline may be absent or found later in old age in Japan (18). Thus, the developmental pattern of self-esteem may differ across cultures, which should be investigated empirically.

### Present Research

To overcome the first limitation of previous research, we measured global self-esteem by administering the Rosenberg Self-Esteem Scale [RSES; (11)]. This scale is one of the most frequently used measures of global self-esteem. We predicted that the developmental trajectory of self-esteem would be consistent with that of self-liking: levels of self-esteem were low in adolescence, but then continued to become higher among adults. Here, we also empirically examined whether self-competence and self-liking were closely related to each other. We expected that self-competence and self-liking would be highly correlated with each other, and the developmental pattern of self-competence would be consistent with that of self-liking. To overcome the second limitation, we collected data covering a more diverse sample that included the elderly over the age of 70. We predicted that a drop of self-esteem would be absent or less sharp in Japanese older adults. In sum, in the current research, we investigated age differences in global self-esteem among a broader range of the population in Japan by using the RSES.

Prior research has shown that the pattern of age differences in self-esteem is similar between males and females in the U.S. [for a review see, Orth and Robins (2)] and Germany (7). Although the patterns are consistent between gender, in some cases, small differences were found with females showing larger age differences than males (4, 5). This was also the case in Japan (19). Thus, we also investigated whether age differences in self-esteem are moderated by gender. We predicted an absence of the moderating effect of gender, but if there were any differences, they would be small differences, which would be larger in females than in males.

## Method

We analyzed six independent web surveys administered to a large and diverse sample in Japan.

### Survey

Each survey was conducted independently in 2009, 2011, 2012, 2013, 2017, and 2018. The data in 2009 was collected by Kyoto University Global COE Program (Revitalizing Education for Dynamic Hearts and Minds). The data for this secondary analysis, “International Comparative Research on Sense of Happiness, 2009–2011” was provided by the Social Science Japan Data Archive (Center for Social Research and Data Archives, Institute of Social Science, The University of Tokyo). The data from 2011, 2012, 2013, 2017, and 2018 were collected by us [e.g., Ogihara et al. (20)]. We recruited participants from every prefecture in Japan on the internet via research firms. The research firms had their own large pools of participants. In each survey, a designated number of participants were assigned to each cell by age category and gender. Participants were rewarded after answering the survey. The summary of each survey is shown in Table 1.

**Table 1:** Summary of surveys.

**Year**	**Age**	**Sample Size**	**Scale Translation**	**Scale Anchor**	**α (RSES)**	**α (Self-Competence)**	**α (Self-Liking)**
2009	18–84	1,221	A	7-point	0.87	0.78	0.76
2011	20–59	800	A	7-point	0.87	0.81	0.74
2012	22–59	763	A	7-point	0.89	0.84	0.75
2013	16–69	997	A	7-point	0.86	0.79	0.74
2017	16–88	1,001	B	5-point	0.87	0.83	0.70
2018	18–87	1,331	B	5-point	0.87	0.82	0.75

*A: the Japanese translation of the Rosenberg Self-Esteem Scale used in previous research [e.g., Heine et al. (8); Uchida et al. (21)], B: Yamamoto et al. (22)*.

Sample sizes by gender and generation are indicated in Table 2. The sample sizes ranged from 763 to 1,331 and the total sample size was 6,113.

**Table 2:** Sample sizes by gender and generation.

**Year**	**Gender**	**10s**	**20s**	**30s**	**40s**	**50s**	**60s**	**70s**	**80s**	**Total**
2009	Male	26	155	–	195	–	185	33	2	596
	Female	38	167	–	204	–	194	20	2	625
	Total	64	322	–	399	–	379	53	4	1,221
2011	Male	–	100	100	100	100	–	–	–	400
	Female	–	100	100	100	100	–	–	–	400
	Total	–	200	200	200	200	–	–	–	800
2012	Male	–	91	82	97	100	–	–	–	370
	Female	–	93	100	100	100	–	–	–	393
	Total	–	184	182	197	200	–	–	–	763
2013	Male	39	77	101	93	91	98	–	–	499
	Female	37	75	98	92	91	105	–	–	498
	Total	76	152	199	185	182	203	–	–	997
2017	Male	30	44	62	75	90	118	56	20	495
	Female	30	30	66	76	104	111	69	20	506
	Total	60	74	128	151	194	229	125	40	1,001
2018	Male	6	107	117	122	135	103	44	2	636
	Female	5	122	144	128	145	117	31	3	695
	Total	11	229	261	250	280	220	75	5	1,331
Total	Male	101	574	462	682	516	504	133	24	2,996
	Female	110	587	508	700	540	527	120	25	3,117
	Total	211	1,161	970	1,382	1,056	1,031	253	49	6,113

### Self-Esteem

The Rosenberg Self-Esteem Scale, a 10-item measure of global self-esteem [e.g., “I feel that I have a number of good qualities,” “On the whole, I am satisfied with myself.”; (11)], was administered.

In the 2017 and 2018 surveys, the Japanese translation of the RSES from Yamamoto et al. (22) was used (5-point scale; 1: Not applicable−5: Applicable). In the other surveys, a different translation of the RSES that has been used in previous research [e.g., Heine et al. (8); Uchida et al. (21)] was administered (7-point scale; 1: Strongly disagree−7: Strongly agree)^2^. Reliabilities of the RSES in the six surveys were sufficiently high (αs > 0.86; Table 1).

The average scores for self-competence (e.g., “I feel that I have a number of good qualities”; SC1^3^) and self-liking (e.g., “On the whole, I am satisfied with myself.”; SL1^4^) were calculated by averaging the five items of the RSES, as was done in previous research (15). The reliabilities for self-competence (αs > 0.77; Table 1) and self-liking (αs > 0.69; Table 1) were sufficiently high.

### Analysis

**Figure 1:**
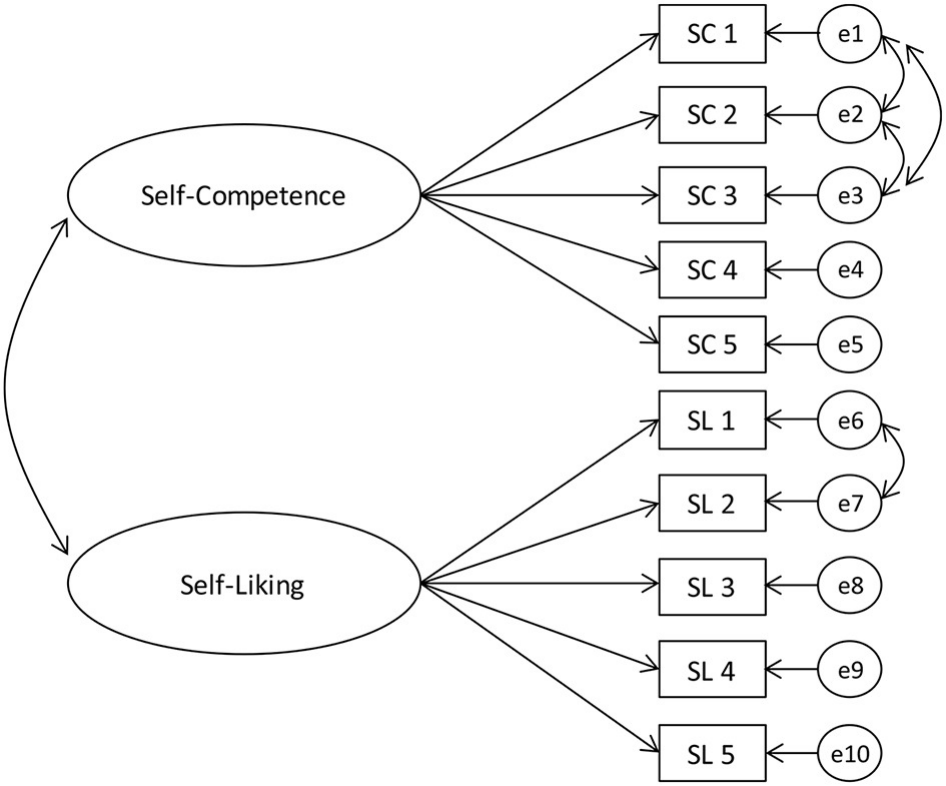
A two-factor model in which self-competence and self-liking constitute self-esteem.

First, we confirmed whether the Self-Esteem Scale measured the same concepts between genders and age-groups (i.e., measurement invariance/equivalence). We divided the participants into subgroups and conducted multi-group confirmatory factor analysis (CFA). For this analysis, we split the participants into three age groups: younger adults (10s^5^, 20s, 30s), middle-aged adults (40s, 50s), and older adults (60s, 70s, 80s)^6^. Following previous research (14, 15), we made a two-factor model in which self-competence (measured by five items) and self-liking (measured by five items) constituted self-esteem (Figure 1)^7^. For the multi-group CFA, we used IBM SPSS Amos (ver. 26).

Then, to check whether self-competence and self-liking are closely related to each other, we calculated their correlation coefficients at the individual level and at the age level.

Next, we conducted hierarchical multiple linear regression analyses on each dataset for predicting self-esteem from age and gender^8^. The independent variables we entered in Step 1 were gender (male = 0, female = 1), the age, and their interaction (age × gender), and in Step 2, the age squared and its interaction with gender (age^2^ × gender), and in Step 3 the age cubed and its interaction with gender (age^3^ × gender). If the interaction effect was significant, we conducted hierarchical multiple linear regression analyses separately by gender for predicting self-esteem from the age (Step 1), the age squared (Step 2), and the age cubed (Step 3). In these analyses, we centered each age variable and weighted sample sizes to estimate the developmental trajectory of self-esteem more precisely.

Finally, we looked at whether these developmental patterns were found in each component (i.e., self-competence and self-liking) by conducting a series of hierarchical multiple linear regression analyses. The dependent variable was the average score for each component (self-competence or self-liking; not global self-esteem). In Step 1, the independent variables were age, the age-squared, and the type of component (categorical variable: self-competence = 0, self-liking = 1). In Step 2, the interaction terms were added (i.e., the age × the component, the age-squared × the component). We used IBM SPSS (ver. 25) for the analyses.

## Results

### Measurement Invariance

We tested measurement invariance in two steps. First, we conducted a confirmatory factor analysis for each subgroup separately (single-group CFA) in each dataset. Second, we conducted confirmatory factor analysis by including the subgroups (multi-group CFA) at four successive levels in each dataset. These results are summarized in Table 3.

**Table 3:** Fit indices for the confirmatory factor analyses.

**Year**	**Group**	**Model**	**χ^2^**	***df***	**SRMR**	**RMSEA [90%CI]**	**CFI**	**Model comparison**	**ΔCFI**
2009	Male	Model 1 (single group)	200.010	30	0.057	0.098 [0.085, 0.111]	0.919	–	–
	Female	Model 1 (single group)	225.450	30	0.069	0.102 [0.090, 0.115]	0.918	–	–
	Male vs. Female	Model 1 (configural invariance)	425.459	60	0.057	0.071 [0.064, 0.077]	0.918	–	–
		Model 2 (metric invariance)	435.555	68	0.063	0.067 [0.061, 0.073]	0.918	2 vs. 1	0.000
		Model 3 (scalar invariance)	452.227	78	0.063	0.063 [0.057, 0.068]	0.916	3 vs. 2	0.002
		Model 4 (structural invariance)	462.640	81	0.065	0.062 [0.057, 0.068]	0.915	4 vs. 3	0.001
	Younger adult	Model 1 (single group)	170.723	30	0.074	0.110 [0.095, 0.127]	0.898	–	–
	Middle-aged adult	Model 1 (single group)	99.772	30	0.052	0.076 [0.060, 0.093]	0.950	–	–
	Older adult	Model 1 (single group)	154.640	30	0.063	0.098 [0.083, 0.113]	0.910	–	–
	Younger adult vs. Middle-aged adult vs. Older adult	Model 1 (configural invariance)	425.137	90	0.074	0.055 [0.050, 0.061]	0.919	–	–
		Model 2 (metric invariance)	449.223	106	0.076	0.052 [0.047, 0.057]	0.917	2 vs. 1	0.002
		Model 3 (scalar invariance)	699.338	126	0.082	0.061 [0.057, 0.066]	0.862	3 vs. 2	0.055
		Model 4 (partial scalar invariance)	494.186	114	0.075	0.052 [0.048, 0.057]	0.908	4 vs. 2	0.009
		Model 5 (structural invariance)	519.227	120	0.090	0.052 [0.048, 0.057]	0.904	5 vs. 4	0.004
2011	Male	Model 1 (single group)	160.192	30	0.061	0.104 [0.089, 0.120]	0.913	–	–
	Female	Model 1 (single group)	155.835	30	0.060	0.103 [0.087, 0.119]	0.930	–	–
	Male vs. Female	Model 1 (configural invariance)	316.027	60	0.061	0.073 [0.065, 0.081]	0.922	–	–
		Model 2 (metric invariance)	334.259	68	0.069	0.070 [0.063, 0.078]	0.919	2 vs. 1	0.003
		Model 3 (scalar invariance)	3520.553	78	0.070	0.066 [0.059, 0.074]	0.916	3 vs. 2	0.003
		Model 4 (structural invariance)	361.397	81	0.072	0.066 [0.059, 0.073]	0.915	4 vs. 3	0.001
	Younger adult	Model 1 (single group)	157.377	30	0.055	0.103 [0.088, 0.119]	0.919	–	–
	Middle-aged adult	Model 1 (single group)	157.216	30	0.069	0.103 [0.088, 0.119]	0.921	–	–
	Younger adult vs. Middle-aged adult	Model 1 (configural invariance)	314.592	60	0.055	0.073 [0.065, 0.081]	0.920	–	–
		Model 2 (metric invariance)	341.546	68	0.067	0.071 [0.064, 0.079]	0.914	2 vs. 1	0.006
		Model 3 (scalar invariance)	378.669	78	0.067	0.070 [0.063, 0.077]	0.905	3 vs. 2	0.009
		Model 4 (structural invariance)	384.037	81	0.069	0.068 [0.062, 0.075]	0.905	4 vs. 3	0.000
2012	Male	Model 1 (single group)	86.613	30	0.045	0.072 [0.054, 0.089]	0.960	–	–
	Female	Model 1 (single group)	182.211	30	0.058	0.114 [0.098, 0.130]	0.926	–	–
	Male vs. Female	Model 1 (configural invariance)	268.817	60	0.045	0.068 [0.060, 0.076]	0.939	–	–
		Model 2 (metric invariance)	281.378	68	0.045	0.064 [0.057, 0.072]	0.938	2 vs. 1	0.001
		Model 3 (scalar invariance)	295.968	78	0.045	0.061 [0.053, 0.068]	0.937	3 vs. 2	0.001
		Model 4 (structural invariance)	306.662	81	0.052	0.061 [0.053, 0.068]	0.935	4 vs. 3	0.002
	Younger adult	Model 1 (single group)	138.903	30	0.058	0.100 [0.083, 0.117]	0.931	–	–
	Middle-aged adult	Model 1 (single group)	130.347	30	0.047	0.092 [0.076, 0.108]	0.943	–	–
	Younger adult vs. Middle-aged adult	Model 1 (configural invariance)	269.253	60	0.058	0.068 [0.060, 0.076]	0.937	–	–
		Model 2 (metric invariance)	279.379	68	0.062	0.064 [0.056, 0.072]	0.937	2 vs. 1	0.000
		Model 3 (scalar invariance)	337.785	78	0.062	0.066 [0.059, 0.073]	0.922	3 vs. 2	0.015
		Model 4 (partial scalar invariance)	316.521	77	0.062	0.064 [0.057, 0.071]	0.928	4 vs. 2	0.009
		Model 5 (structural invariance)	319.233	80	0.061	0.063 [0.056, 0.070]	0.928	5 vs. 4	0.000
2013	Male	Model 1 (single group)	260.438	30	0.092	0.124 [0.111, 0.138]	0.864	–	–
	Female	Model 1 (single group)	322.207	30	0.097	0.140 [0.126, 0.154]	0.880	–	–
	Male vs. Female	Model 1 (configural invariance)	582.645	60	0.092	0.094 [0.087, 0.101]	0.874	–	–
		Model 2 (metric invariance)	597.887	68	0.091	0.088 [0.082, 0.095]	0.872	2 vs. 1	0.002
		Model 3 (scalar invariance)	652.347	78	0.090	0.086 [0.080, 0.092]	0.861	3 vs. 2	0.011
		Model 4 (partial scalar invariance)	625.564	77	0.091	0.085 [0.079, 0.091]	0.867	4 vs. 2	0.005
		Model 5 (structural invariance)	638.282	80	0.096	0.084 [0.078, 0.090]	0.865	5 vs. 4	0.002
	Younger adult	Model 1 (single group)	335.349	30	0.117	0.155 [0.140, 0.170]	0.833	–	–
	Middle-aged adult	Model 1 (single group)	139.292	30	0.067	0.100 [0.083, 0.117]	0.916	–	–
	Older adult	Model 1 (single group)	113.490	30	0.105	0.117 [0.095, 0.141]	0.882	–	–
	Younger adult vs. Middle-aged adult vs. Older adult	Model 1 (configural invariance)	588.085	90	0.117	0.075 [0.069, 0.080]	0.870	–	–
		Model 2 (metric invariance)	607.223	106	0.116	0.069 [0.064, 0.074]	0.869	2 vs. 1	0.001
		Model 3 (scalar invariance)	727.020	126	0.114	0.069 [0.064, 0.074]	0.843	3 vs. 2	0.026
		Model 4 (partial scalar invariance)	656.256	114	0.114	0.069 [0.064, 0.074]	0.859	4 vs. 2	0.010
		Model 5 (structural invariance)	677.391	120	0.119	0.068 [0.063, 0.073]	0.855	5 vs. 4	0.004
2017	Male	Model 1 (single group)	134.581	30	0.055	0.084 [0.070, 0.099]	0.954	–	–
	Female	Model 1 (single group)	235.731	30	0.060	0.117 [0.103, 0.131]	0.923	–	–
	Male vs. Female	Model 1 (configural invariance)	370.310	60	0.055	0.072 [0.065, 0.079]	0.937	–	–
		Model 2 (metric invariance)	386.446	68	0.061	0.068 [0.062, 0.075]	0.935	2 vs. 1	0.002
		Model 3 (scalar invariance)	415.211	78	0.060	0.066 [0.060, 0.072]	0.931	3 vs. 2	0.004
		Model 4 (structural invariance)	416.400	81	0.062	0.064 [0.058, 0.071]	0.932	4 vs. 3	0.001
	Younger adult	Model 1 (single group)	142.153	30	0.073	0.120 [0.100, 0.140]	0.920	–	–
	Middle-aged adult	Model 1 (single group)	140.548	30	0.061	0.103 [0.087, 0.121]	0.930	–	–
	Older adult	Model 1 (single group)	90.967	30	0.049	0.072 [0.055, 0.089]	0.954	–	–
	Younger adult vs. Middle-aged adult vs. Older adult	Model 1 (configural invariance)	373.730	90	0.073	0.056 [0.050, 0.062]	0.935	–	–
		Model 2 (metric invariance)	404.120	106	0.079	0.053 [0.048, 0.059]	0.931	2 vs. 1	0.004
		Model 3 (scalar invariance)	597.741	126	0.088	0.061 [0.056, 0.066]	0.891	3 vs. 2	0.040
		Model 4 (partial scalar invariance)	462.783	110	0.079	0.057 [0.051, 0.062]	0.919	4 vs. 2	0.012
		Model 5 (structural invariance)	440.827	112	0.109	0.054 [0.049, 0.060]	0.924	5 vs. 2	0.007
2018	Male	Model 1 (single group)	272.000	30	0.083	0.113 [0.101, 0.125]	0.922	–	–
	Female	Model 1 (single group)	258.082	30	0.072	0.105 [0.093, 0.117]	0.935	–	–
	Male vs. Female	Model 1 (configural invariance)	530.085	60	0.083	0.077 [0.071, 0.083]	0.929	–	–
		Model 2 (metric invariance)	550.419	68	0.087	0.073 [0.067, 0.079]	0.927	2 vs. 1	0.002
		Model 3 (scalar invariance)	601.791	78	0.086	0.071 [0.066, 0.076]	0.921	3 vs. 2	0.006
		Model 4 (structural invariance)	603.280	81	0.085	0.070 [0.065, 0.075]	0.921	4 vs. 3	0.000
	Younger adult	Model 1 (single group)	325.514	30	0.107	0.140 [0.127, 0.154]	0.883	–	–
	Middle-aged adult	Model 1 (single group)	161.942	30	0.063	0.091 [0.078, 0.105]	0.942	–	–
	Older adult	Model 1 (single group)	93.372	30	0.064	0.084 [0.065, 0.104]	0.948	–	–
	Younger adult vs. Middle-aged adult vs. Older adult	Model 1 (configural invariance)	580.786	90	0.107	0.064 [0.059, 0.069]	0.919	–	–
		Model 2 (metric invariance)	617.604	106	0.107	0.060 [0.056, 0.065]	0.915	2 vs. 1	0.004
		Model 3 (scalar invariance)	794.764	126	0.105	0.063 [0.059, 0.067]	0.889	3 vs. 2	0.026
		Model 4 (partial scalar invariance)	681.359	110	0.107	0.063 [0.058, 0.067]	0.905	4 vs. 2	0.010
		Model 5 (structural invariance)	716.654	116	0.124	0.062 [0.058, 0.067]	0.900	5 vs. 4	0.005

*df, degree of freedom; SRMR, standardized root mean square residual; RMSEA, root mean square error of approximation; CI, confidence interval; CFI, comparative fit index*.

#### Single-Group CFA

The model fits were acceptable to adequate in all the datasets (Table 3), showing that the two-factor model successfully described the construction of self-esteem in each subgroup.

#### Multi-Group CFA

To evaluate the fitness of the model at four hierarchical levels, we used changes in CFI (ΔCFI) index. Specifically, if ΔCFI was smaller than 0.010, the successive model that constrained more equality was accepted (27). We did not use Δχ^2^ for this evaluation because χ^2^ is sensitive to sample size (27).

First, configural invariance was tested. Configural invariance indicates that structure of latent factors and observed variables (e.g., number of latent factors, same associations between each factor and observed items) are consistent across groups. Second, metric invariance (weak factorial invariance) was investigated. Metric invariance means that factor loadings are comparable across groups, indicating that participants in different groups respond to each item in a similar way. We constrained each factor loading to be equal across groups. Third, scalar invariance (strong factorial invariance) was tested. Scalar invariance indicates that factor loadings and intercepts of items are comparable across groups, showing that latent factor scores lead to observed scores in the same way across groups. We constrained each factor loading and intercept of each observed variable to be equal across groups. Finally, structural invariance (factor variance/covariance invariance) was examined. Structural invariance means that latent factors are distributed and associated similarly across groups. We constrained the variance of each factor and covariance of the two factors across groups.

Regarding gender, five out of the six datasets (2009, 2011, 2012, 2017, 2018 datasets) demonstrated scalar and structural invariance, and one (2013 dataset) showed partial scalar^9^ and structural invariance. In the 2013 dataset, ΔCFI between the metric invariance model and the scalar invariance model was 0.011, which was slightly above the conventional criterion of 0.010 (27). This criterion is not a golden rule, and excluding only one constraint of item intercept cleared the criterion (partial scalar invariance). In all of the datasets, at least partial scalar and structural invariance were supported.

Regarding age-group, one out of the six datasets (2011 dataset) showed scalar and structural invariance, four (2009, 2012, 2013, 2018 datasets) showed partial scalar^10^ and structural invariance, one (2017 dataset) showed metric and factorial invariance^11^. In the 2017 dataset, ΔCFI between the metric invariance model and the partial scalar invariance model was 0.012, which was slightly above the conventional criterion of 0.010 (27). Overall, in the most datasets, at least partial scalar and structural invariance were supported.

### The Relationship Between Self-Competence and Self-Liking

We calculated correlation coefficients between self-competence and self-liking at the individual level and at the age level in each of the six studies (Table 4). At the individual level, they were highly correlated for the total population (*r*s > 0.72, *p*s < 0.001), for males (*r*s > 0.68, *p*s < 0.001), and for females (*r*s > 0.72, *p*s < 0.001). These strong relationships were also found within sub-populations (each age group for both males and females). Relatively lower coefficients for some sub-populations (*r* = 0.42 for males in their 70s in 2018, *r* = 0.27 for females in their teens in 2018) were due to the small sample sizes (*n* = 44 for males in their 70s in 2018, *n* = 5 for females in their teens in 2018; see Table 2). Similarly, at the age level, the correlations were large both for males (*r*s > 0.71, *p*s < 0.001) and females (*r*s > 0.79, *p*s < 0.001).

**Table 4:** Correlation coefficients between self-competence and self-liking.

	**Individual level**																			**Age level**	
	**Total**	**Male**									**Female**									**Male**	**Female**
**Year**		**Total**	**10s**	**20s**	**30s**	**40s**	**50s**	**60s**	**70s**	**80s**	**Total**	**10s**	**20s**	**30s**	**40s**	**50s**	**60s**	**70s**	**80s**		
2009	0.75^***^	0.77^***^	0.85^***^	0.77^***^	–	0.77^***^	–	0.70^***^	0.85^***^	–	0.73^***^	0.79^***^	0.68^***^	–	0.75^***^	–	0.73^***^	0.82^***^	–	0.92^***^	0.80^***^
2011	0.78^***^	0.78^***^	–	0.75^***^	0.83^***^	0.77^***^	0.77^***^	–	–	–	0.78^***^	–	0.77^***^	0.77^***^	0.70^***^	0.78^***^	–	–	–	0.72^***^	0.87^***^
2012	0.78^***^	0.74^***^	–	0.76^***^	0.64^***^	0.84^***^	0.67^***^	–	–	–	0.81^***^	–	0.82^***^	0.77^***^	0.80^***^	0.81^***^	–	–	–	0.75^***^	0.89^***^
2013	0.74^***^	0.69^***^	0.81^***^	0.60^***^	0.70^***^	0.61^***^	0.70^***^	0.68^***^	–	–	0.77^***^	0.77^***^	0.72^***^	0.75^***^	0.79^***^	0.75^***^	0.74^***^	–	–	0.73^***^	0.85^***^
2017	0.77^***^	0.75^***^	0.92^***^	0.77^***^	0.76^***^	0.68^***^	0.52^***^	0.74^***^	0.68^***^	0.53^*^	0.79^***^	0.67^***^	0.88^***^	0.79^***^	0.82^***^	0.73^***^	0.77^***^	0.70^***^	0.81^***^	0.86^***^	0.88^***^
2018	0.73^***^	0.72^***^	0.96^**^	0.78^***^	0.70^***^	0.61^***^	0.76^***^	0.66^***^	0.42^**^	–	0.74^***^	0.27	0.78^***^	0.70^***^	0.70^***^	0.66^***^	0.67^***^	0.68^***^	0.95	0.81^***^	0.79^***^

*** *p < 0.001*,

** *p < 0.01*,

* *p < 0.05*.

### Age Differences in Global Self-Esteem

A summary of the regression models predicting self-esteem from age and gender is indicated in Table 5.

**Table 5:** Summary of regression models predicting self-esteem from age and gender.

		**Step 1**					**Step 2**					**Step 3**				
**Year**		***B***	***SE***	**95% *CI***	**β**	***p***	***B***	***SE***	**95% *CI***	**β**	***p***	***B***	***SE***	**95% *CI***	**β**	***p***
2009	Gender	0.01	0.04	[−0.07, 0.09]	0.02	0.76	0.11	0.06	[−0.01, 0.23]	0.22	0.07	0.12	0.07	[−0.01, 0.25]	0.23	0.08
	Age	0.009	0.002	[0.005, 0.012]	0.62	^***^	0.009	0.001	[0.006, 0.012]	0.63	^***^	0.01	0.005	[0.001, 0.020]	0.77	^*^
	Age × Gender	0.003	0.002	[−0.002, 0.007]	0.15	0.21	0.003	0.002	[−0.001, 0.007]	0.15	0.18	0.001	0.007	[−0.01, 0.01]	0.07	0.84
	Age^2^						0.0004	0.0001	[0.0001, 0.0006]	0.37	^**^	0.0004	0.0001	[0.0001, 0.0006]	0.38	^**^
	Age^2^ × Gender						−0.0003	0.0001	[−0.00059, −0.00001]	−0.29	^*^	−0.0003	0.0002	[−0.000630, 0.000003]	−0.31	0.05
	Age^3^											−0.000004	0.00001	[−0.00002, 0.00001]	−0.14	0.68
	Age^3^ × Gender											0.000003	0.00001	[−0.00002, 0.00003]	0.08	0.81
	Δ*R*^2^									0.07	^**^				0.001	0.92
	*R*^2^				0.54	^***^				0.60	^***^				0.61	^***^
2011	Gender	−0.06	0.06	[−0.18, 0.07]	−0.08	0.38	−0.04	0.10	[−0.24, 0.15]	−0.06	0.65	−0.04	0.10	[−0.23, 0.16]	−0.05	0.71
	Age	0.01	0.004	[0.002, 0.019]	0.33	^*^	0.01	0.004	[0.003, 0.019]	0.33	^*^	0.02	0.01	[−0.004, 0.035]	0.49	0.11
	Age × Gender	0.02	0.006	[0.004, 0.028]	0.34	^*^	0.02	0.006	[0.003, 0.027]	0.32	^*^	−0.001	0.01	[−0.03, 0.03]	−0.01	0.96
	Age^2^						0.001	0.0004	[−0.0003, 0.0014]	0.17	0.18	0.001	0.0004	[−0.0003, 0.0014]	0.17	0.18
	Age^2^ × Gender						−0.0001	0.001	[−0.001, 0.001]	−0.02	0.89	−0.0002	0.001	[−0.001, 0.001]	−0.05	0.74
	Age^3^											−0.00002	0.00004	[−0.0001, 0.0001]	−0.17	0.56
	Age^3^ × Gender											0.0001	0.00006	[−0.00005, 0.00020]	0.37	0.22
	Δ*R*^2^									0.02	0.24				0.01	0.45
	*R*^2^				0.38	^***^				0.40	^***^				0.42	^***^
2012	Gender	−0.03	0.08	[−0.19, 0.14]	−0.03	0.75	0.10	0.13	[−0.15, 0.35]	0.11	0.44	0.10	0.13	[−0.15, 0.36]	0.12	0.42
	Age	0.02	0.01	[0.01, 0.03]	0.43	^**^	0.02	0.01	[0.01, 0.03]	0.42	^**^	0.01	0.01	[−0.01, 0.04]	0.32	0.34
	Age × Gender	0.01	0.01	[−0.003, 0.029]	0.22	0.11	0.01	0.01	[−0.002, 0.030]	0.24	0.09	0.01	0.02	[−0.03, 0.04]	0.09	0.80
	Age^2^						0.001	0.001	[−0.001, 0.002]	0.16	0.25	0.001	0.001	[−0.001, 0.002]	0.15	0.29
	Age^2^ × Gender						−0.001	0.001	[−0.003, 0.001]	−0.22	0.20	−0.001	0.001	[−0.003, 0.001]	−0.25	0.16
	Age^3^											0.00002	0.0001	[−0.0001, 0.0002]	0.11	0.74
	Age^3^ × Gender											0.00005	0.0001	[−0.0001, 0.0002]	0.17	0.62
	Δ*R*^2^									0.02	0.41				0.01	0.57
	*R*^2^				0.37	^***^				0.39	^***^				0.40	^***^
2013	Gender	−0.04	0.05	[−0.15, 0.06]	−0.06	0.40	−0.01	0.08	[−0.17, 0.14]	−0.02	0.85	−0.02	0.08	[−0.17, 0.13]	−0.03	0.80
	Age	0.01	0.002	[0.01, 0.02]	0.50	^***^	0.01	0.002	[0.01, 0.02]	0.50	^***^	0.02	0.01	[0.01, 0.03]	0.67	^**^
	Age × Gender	0.01	0.003	[0.003, 0.017]	0.28	^**^	0.01	0.004	[0.003, 0.017]	0.27	^**^	0.01	0.01	[−0.01, 0.02]	0.17	0.47
	Age^2^						−0.00002	0.0002	[−0.0004, 0.0003]	−0.01	0.92	−0.0001	0.0002	[−0.0004, 0.0003]	−0.03	0.77
	Age^2^ × Gender						−0.0001	0.0003	[−0.0006, 0.0004]	−0.06	0.60	−0.0001	0.0003	[−0.0006, 0.0004]	−0.05	0.70
	Age^3^											−0.00001	0.00001	[−0.00004, 0.00002]	−0.18	0.44
	Age^3^ × Gender											0.00001	0.00002	[−0.00003, 0.00005]	0.11	0.64
	Δ*R*^2^									0.003	0.71				0.003	0.74
	*R*^2^				0.53	^***^				0.53	^***^				0.54	^***^
2017	Gender	−0.001	0.04	[−0.08, 0.08]	0.00	0.99	0.03	0.05	[−0.08, 0.14]	0.04	0.57	0.03	0.05	[−0.08, 0.14]	0.04	0.58
	Age	0.02	0.002	[0.01, 0.02]	0.77	^***^	0.02	0.002	[0.01, 0.02]	0.83	^***^	0.02	0.003	[0.01, 0.02]	0.91	^***^
	Age × Gender	−0.001	0.002	[−0.005, 0.004]	−0.02	0.82	−0.001	0.002	[−0.006, 0.003]	−0.04	0.62	−0.002	0.005	[−0.01, 0.01]	−0.06	0.73
	Age^2^						0.0002	0.0001	[0.00003, 0.00037]	0.19	^*^	0.0002	0.0001	[−0.000003, 0.000365]	0.17	0.05
	Age^2^ × Gender						−0.0001	0.0001	[−0.0003, 0.0001]	−0.07	0.44	−0.0001	0.0001	[−0.0004, 0.0002]	−0.07	0.50
	Age^3^											−0.000003	0.000005	[−0.00001, 0.00001]	−0.10	0.59
	Age^3^ × Gender											0.000001	0.00001	[−0.00001, 0.00001]	0.02	0.91
	Δ*R*^2^									0.02	^*^				0.001	0.80
	*R*^2^				0.57	^***^				0.59	^***^				0.59	^***^
2018	Gender	0.08	0.04	[0.01, 0.16]	0.13	^*^	0.12	0.05	[0.01, 0.23]	0.19	^*^	0.12	0.05	[0.01, 0.23]	0.18	^*^
	Age	0.01	0.002	[0.01, 0.02]	0.68	^***^	0.01	0.002	[0.01, 0.02]	0.67	^***^	0.02	0.004	[0.01, 0.03]	0.82	^***^
	Age × Gender	0.003	0.003	[−0.002, 0.008]	0.09	0.27	0.003	0.003	[−0.002, 0.008]	0.10	0.24	0.004	0.01	[−0.01, 0.01]	0.12	0.48
	Age^2^						0.0001	0.0001	[−0.0001, 0.0003]	0.07	0.43	0.0001	0.0001	[−0.0001, 0.0004]	0.09	0.29
	Age^2^ × Gender						−0.0002	0.0002	[−0.0005, 0.0002]	−0.10	0.30	−0.0002	0.0002	[−0.0005, 0.0002]	−0.10	0.37
	Age^3^											−0.00001	0.00001	[−0.00002, 0.00001]	−0.17	0.36
	Age^3^ × Gender											−0.000002	0.00001	[−0.00002, 0.00002]	−0.03	0.86
	Δ*R*^2^									0.004	0.58				0.01	0.29
	*R*^2^				0.57	^***^				0.58	^***^				0.58	^***^

*** *p < 0.001*,

** *p < 0.01*,

* *p < 0.05*.

*Gender was coded as male = 0, female = 1*.

#### 2009

The model in Step 1 was significant, and the addition of the age squared and its interaction with gender significantly increased the coefficient of determination (Step 2; Table 5). The addition of the age cubed and its interaction with gender did not significantly increase the coefficient of determination (Step 3). Thus, the quadratic model (Step 2) was accepted. We conducted a consistent analysis separately by gender because the interaction between the age squared and gender was significant.

##### Male

**Figure 2:**
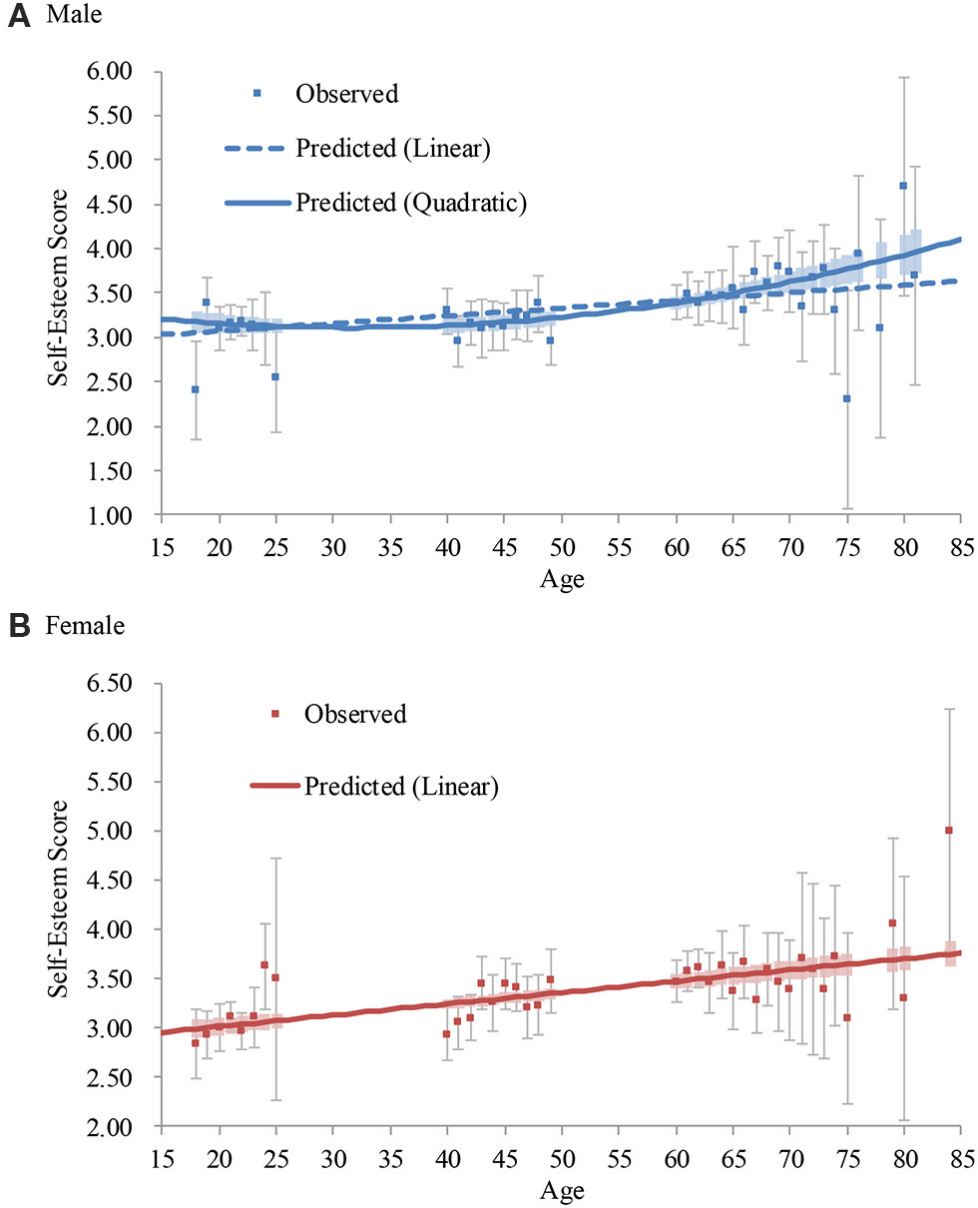
Average and predicted self-esteem scores across ages in Japan (2009 survey). Note. Error bars represent 95% confidence intervals.

The age significantly predicted self-esteem (Step 1), and the addition of the age squared term significantly increased the coefficient of determination (Step 2; Table 6). The addition of the age cubed term did not significantly increase the coefficient of determination (Step 3). Thus, the quadratic model (Step 2) was accepted (Figure 2). Self-esteem showed a slight downward trend from the teens to the mid-30s (the lowest predicted score was the age of 32^12^), but then it continued to become higher to the 80s^13^.

**Table 6:** Summary of regression models predicting self-esteem from age by gender (in the 2009, 2011, and 2013 surveys).

			**Step 1**					**Step 2**					**Step 3**				
**Year**	**Gender**		***B***	***SE***	**95% *CI***	**β**	***p***	***B***	***SE***	**95% *CI***	**β**	***p***	***B***	***SE***	**95% *CI***	**β**	***p***
2009	Male	Age	0.01	0.002	[0.01, 0.01]	0.65	^***^	0.01	0.001	[0.01, 0.01]	0.70	^***^	0.01	0.005	[0.001, 0.021]	0.84	^*^
		Age^2^						0.0004	0.0001	[0.0001, 0.0006]	0.39	^**^	0.0004	0.0001	[0.0001, 0.006]	0.39	^**^
		Age^3^											−0.000004	0.00001	[−0.00002, 0.00001]	−0.15	0.68
		Δ*R*^2^									0.15	^**^				0.002	0.68
		*R*^2^				0.42	^***^				0.57	^***^				0.57	^***^
	Female	Age	0.01	0.001	[0.01, 0.01]	0.80	^***^	0.01	0.001	[0.01, 0.01]	0.80	^***^	0.01	0.005	[0.003, 0.021]	0.83	^*^
		Age^2^						0.0001	0.0001	[−0.0002, 0.0003]	0.05	0.62	0.0001	0.0001	[−0.0002, 0.0003]	0.06	0.63
		Age^3^											−0.000001	0.00001	[−0.00002, 0.00002]	−0.03	0.93
		Δ*R*^2^									0.003	0.62				0.0001	0.93
		*R*^2^				0.63	^***^				0.64	^***^				0.64	^***^
2011	Male	Age	0.01	0.004	[0.002, 0.019]	0.37	^*^	0.01	0.004	[0.002, 0.020]	0.38	^*^	0.02	0.01	[−0.005, 0.037]	0.56	0.12
		Age^2^						0.001	0.0004	[−0.0003, 0.0015]	0.19	0.20	0.001	0.0004	[−0.0003, 0.0015]	0.19	0.21
		Age^3^											−0.00002	0.00004	[−0.0001, 0.0001]	−0.20	0.58
		Δ*R*^2^									0.04	0.20				0.01	0.58
		*R*^2^				0.14	^*^				0.17	^*^				0.18	0.06
	Female	Age	0.03	0.004	[0.02, 0.03]	0.73	^***^	0.03	0.004	[0.02, 0.03]	0.71	^***^	0.02	0.01	[−0.005, 0.035]	0.42	0.13
		Age^2^						0.0005	0.0004	[−0.0004, 0.0014]	0.12	0.28	0.0003	0.0005	[−0.001, 0.001]	0.09	0.44
		Age^3^											0.0001	0.00004	[−0.00004, 0.00014]	0.33	0.24
		Δ*R*^2^									0.02	0.28				0.02	0.24
		*R*^2^				0.53	^***^				0.55	^***^				0.56	^***^
2013	Male	Age	0.01	0.003	[0.01, 0.02]	0.58	^***^	0.01	0.003	[0.01, 0.02]	0.58	^***^	0.02	0.01	[0.005, 0.030]	0.77	^**^
		Age^2^						−0.00002	0.0002	[−0.0004, 0.0003]	−0.01	0.92	−0.00005	0.0002	[−0.0004, 0.0003]	−0.03	0.79
		Age^3^											−0.00001	0.00001	[−0.00004, 0.00002]	−0.21	0.45
		Δ*R*^2^									0.0001	0.92				0.01	0.45
		*R*^2^				0.34	^***^				0.34	^***^				0.35	^***^
	Female	Age	0.02	0.002	[0.02, 0.03]	0.80	^***^	0.02	0.002	[0.02, 0.03]	0.79	^***^	0.02	0.01	[0.01, 0.04]	0.81	^***^
		Age^2^						−0.0001	0.0002	[−0.0005, 0.0002]	−0.07	0.40	−0.0002	0.0002	[−0.0005, 0.0002]	−0.07	0.41
		Age^3^											−0.000001	0.00001	[−0.00003, 0.00003]	−0.02	0.93
		Δ*R*^2^									0.01	0.40				0.0001	0.93
		*R*^2^				0.64	^***^				0.65	^***^				0.65	^***^

*** *p < 0.001*,

** *p < 0.01*,

* *p < 0.05*.

##### Female

The model in Step 1 was significant, and the addition of the age squared term did not significantly increase the coefficient of determination (Step 2; Table 6). Thus, the linear model (Step 1) was accepted (Figure 2). Self-esteem continued to become higher from the teens to the 80s (*d* = 0.97^14^)^15^.

#### 2011

The model in Step 1 was significant, and the addition of the age squared and its interaction with gender did not significantly increase the coefficient of determination (Step 2; Table 5). Thus, the linear model (Step 1) was accepted. We conducted a consistent analysis separately by gender because the interaction between the age and gender was significant.

##### Male

**Figure 3:**
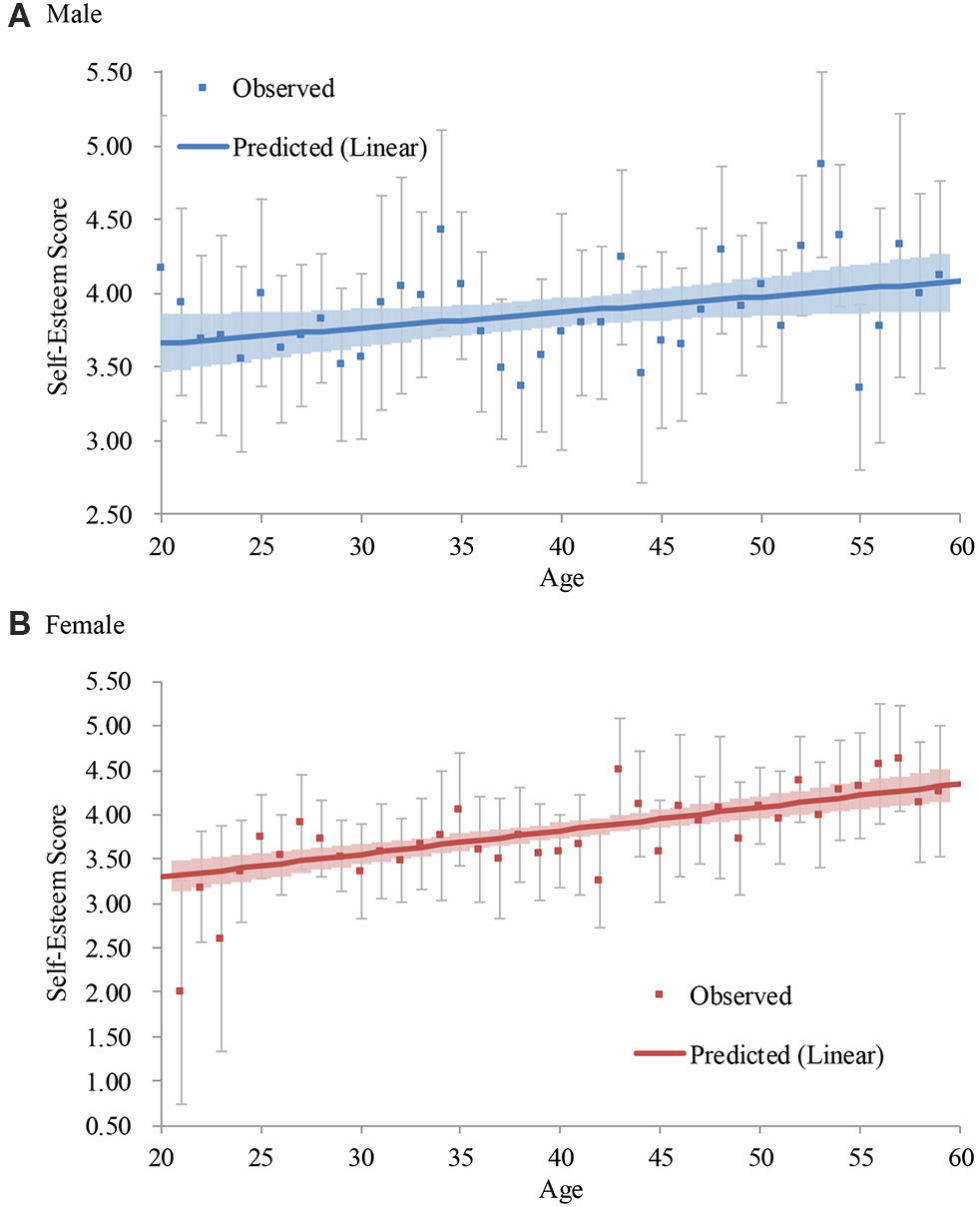
Average and predicted self-esteem scores across ages in Japan (2011 survey). Note. Error bars represent 95% confidence intervals.

The model in Step 1 was significant, and the addition of the age squared term did not significantly increase the coefficient of determination (Step 2; Table 6). Thus, the linear model (Step 1) was accepted (Figure 3). Self-esteem continued to become higher from the 20s to the 50s (*d* = 0.51).

##### Female

The model in Step 1 was significant, and the addition of the age squared term did not significantly increase the coefficient of determination (Step 2; Table 6). Thus, the linear model (Step 1) was accepted (Figure 3). Self-esteem continued to become higher from the 20s to the 50s (*d* = 1.05). The slope for females (*B* = 0.03, *p* < 0.001) was larger than that for males (*B* = 0.01, *p* < 0.05).

#### 2012

**Figure 4:**
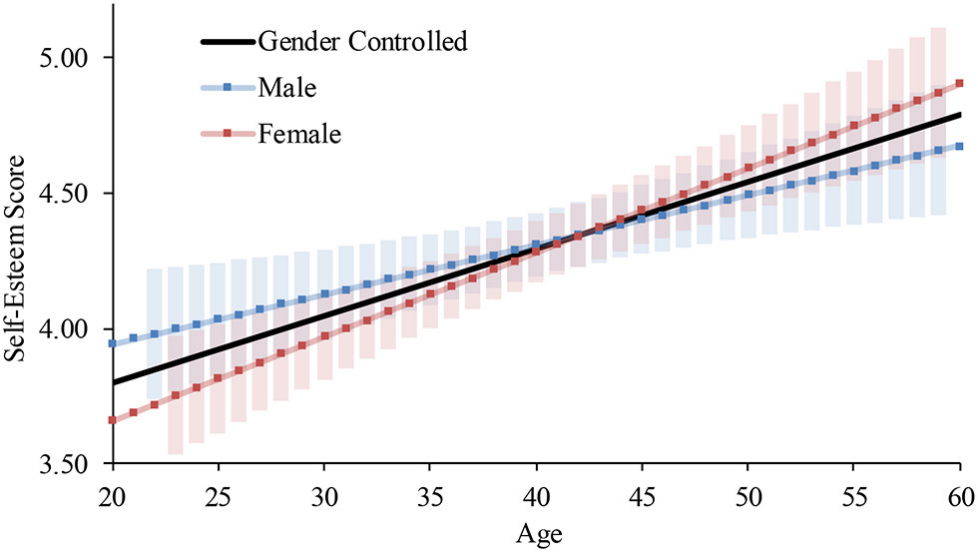
Predicted self-esteem scores across ages in Japan (2012 survey). Note. Error bars represent 95% confidence intervals.

The model in Step 1 was significant, and the addition of the age squared and its interaction with gender did not significantly increase the coefficient of determination (Step 2; Table 5). Thus, the linear model (Step 1) was accepted. The interaction between the age and gender was not significant, showing that self-esteem continued to become higher from the 20s to the 50s both for males and females (Figure 4)^16^.

#### 2013

The model in Step 1 was significant, and the addition of the age squared and its interaction with gender did not significantly increase the coefficient of determination (Step 2; Table 5). Thus, the linear model (Step 1) was accepted. We conducted a consistent analysis separately by gender because the interaction between the age and gender was significant.

##### Male

**Figure 5:**
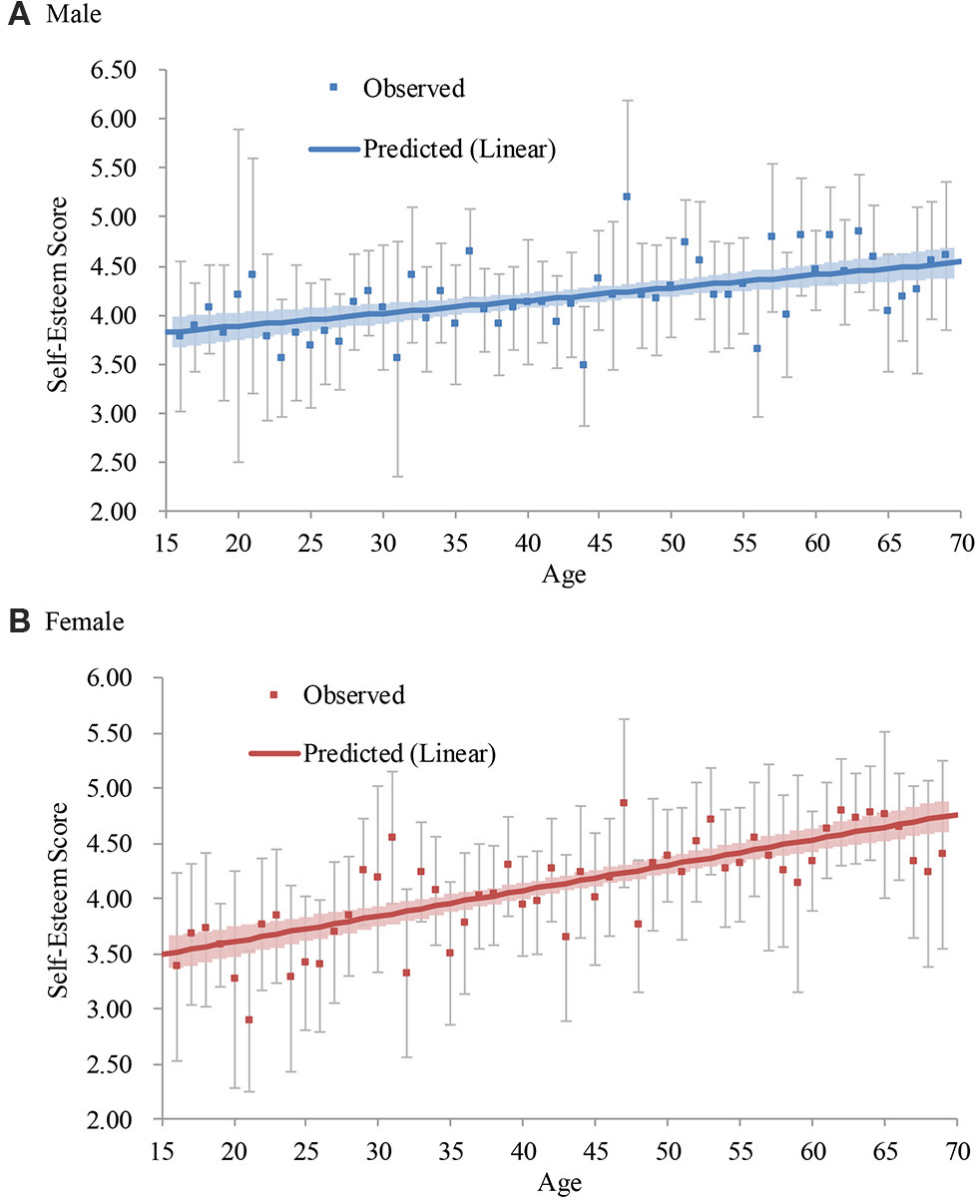
Average and predicted self-esteem scores across ages in Japan (2013 survey). Note. Error bars represent 95% confidence intervals.

The model in Step 1 was significant, and the addition of the age squared term did not significantly increase the coefficient of determination (Step 2; Table 6). Thus, the linear model (Step 1) was accepted (Figure 5). Self-esteem continued to become higher from the teens to the 60s (*d* = 1.00).

##### Female

The model in Step 1 was significant, and the addition of the age squared term did not significantly increase the coefficient of determination (Step 2; Table 6). Thus, the linear model (Step 1) was accepted (Figure 5). Self-esteem continued to become higher from the teens to the 60s (*d* = 1.42). The slope for females (*B* = 0.02, *p* < 0.001) was larger than that for males (*B* = 0.01, *p* < 0.001).

#### 2017

**Figure 6:**
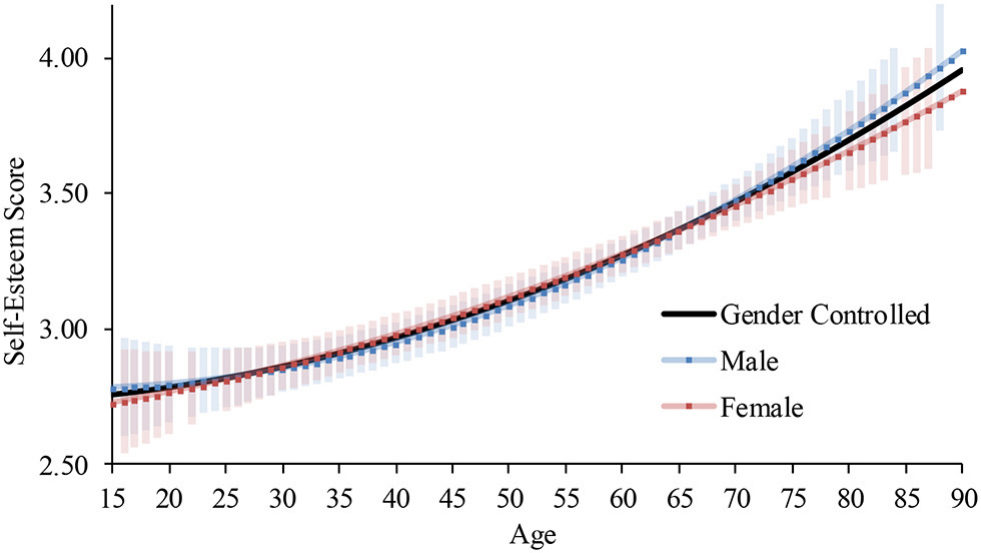
Predicted self-esteem scores across ages in Japan (2017 survey). Error bars represent 95% confidence intervals.

The model in Step 1 was significant, and the addition of the age squared and its interaction with gender significantly increased the coefficient of determination (Step 2; Table 5). The addition of the age cubed and its interaction with gender did not significantly increase the coefficient of determination (Step 3). Thus, the quadratic model (Step 2) was accepted. The interaction between the age squared and gender was not significant. Self-esteem continued to become higher from the 20s to the 50s both for males and females (Figure 6)^17^.

#### 2018

**Figure 7:**
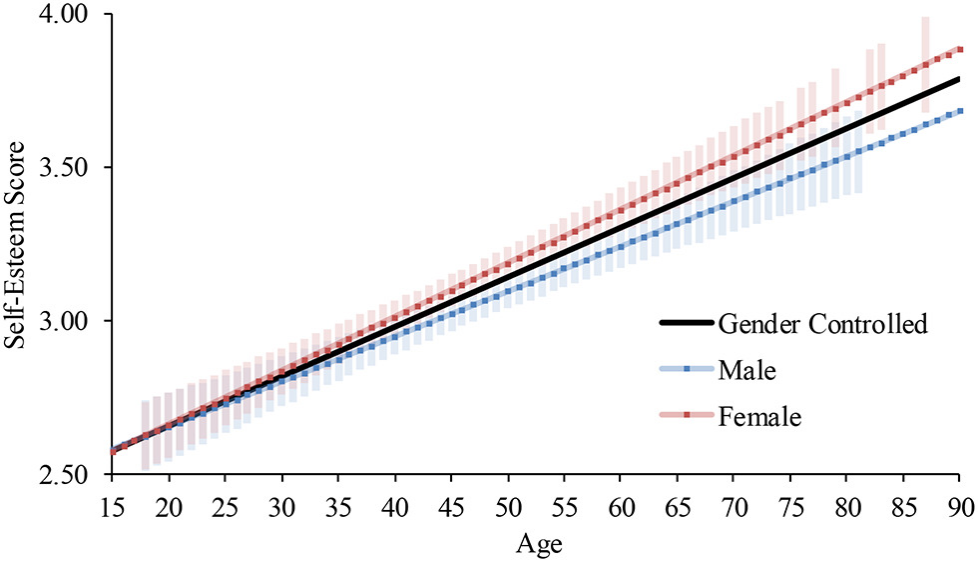
Predicted self-esteem scores across ages in Japan (2018 survey). Note. Error bars represent 95% confidence intervals.

The model in Step 1 was significant, and the addition of the age squared and its interaction with gender did not significantly increase the coefficient of determination (Step 2; Table 5). Thus, the linear model (Step 1) was accepted. The interaction between the age and gender was not significant, showing that self-esteem continued to become higher from the 20s to the 50s both for males and females (Figure 7)^18^.

### Age Differences in Self-Competence and Self-Liking (The Two Components of Global Self-Esteem)

The results of the hierarchical multiple regression analyses are summarized in Table 7. Except for females in 2009, the additions of their interaction terms did not significantly increase the coefficient of determination. Neither of the interaction terms significantly predicted the scores. These results consistently suggest that the developmental patterns for self-competence and self-liking were not different.

**Table 7:** Hierarchical multiple linear regression predicting components of self-esteem from age, component type and their interactions.

			**Step 1**					**Step 2**				
**Year**	**Gender**		***B***	***SE***	**95% *CI***	**β**	***p***	***B***	***SE***	**95% *CI***	**β**	***p***
2009	Male	Age	0.01	0.001	[0.01, 0.01]	0.68	^***^	0.01	0.002	[0.005, 0.011]	0.56	^***^
		Age^2^	0.0004	0.0001	[0.0002, 0.0005]	0.37	^***^	0.0003	0.0001	[0.0001, 0.0005]	0.32	^**^
		Component	−0.03	0.04	[−0.11, 0.05]	−0.07	0.42	−0.07	0.06	[−0.19, 0.06]	−0.13	0.31
		Age × Component						0.003	0.002	[−0.001, 0.008]	0.16	0.16
		Age^2^ × Component						0.0001	0.0002	[−0.0002, 0.0004]	0.10	0.51
		Δ*R*^2^									0.01	0.33
		*R*^2^				0.53	^***^				0.55	^***^
	Female	Age	0.01	0.001	[0.01, 0.01]	0.73	^***^	0.01	0.002	[0.004, 0.011]	0.47	^***^
		Age^2^	0.0001	0.0001	[−0.0001, 0.0002]	0.05	0.55	0.0002	0.0001	[−0.00005, 0.00040]	0.16	0.12
		Component	−0.12	0.04	[−0.21, −0.03]	−0.21	^**^	−0.04	0.07	[−0.17, 0.09]	–0.07	0.55
		Age × Component						0.01	0.002	[0.004, 0.013]	0.36	^***^
		Age^2^ × Component						−0.0002	0.0002	[−0.0006, 0.0001]	−0.21	0.12
		Δ*R*^2^									0.08	^***^
		*R*^2^				0.57	^***^				0.65	^***^
2011	Male	Age	0.01	0.003	[0.01, 0.02]	0.38	^***^	0.01	0.005	[0.001, 0.020]	0.33	^*^
		Age^2^	0.001	0.0003	[0.00003, 0.00137]	0.21	^*^	0.001	0.0005	[−0.0002, 0.0017]	0.22	0.14
		Component	−0.08	0.07	[−0.22, 0.06]	−0.11	0.27	−0.08	0.11	[−0.29, 0.14]	−0.11	0.49
		Age × Component						0.004	0.01	[−0.01, 0.02]	0.08	0.59
		Age^2^ × Component						−0.00003	0.001	[−0.001, 0.001]	−0.01	0.96
		Δ*R*^2^									0.003	0.86
		*R*^2^				0.20	^**^				0.20	^**^
	Female	Age	0.03	0.003	[0.02, 0.04]	0.69	^***^	0.03	0.005	[0.02, 0.04]	0.67	^***^
		Age^2^	0.001	0.0004	[−0.0001, 0.0013]	0.13	0.11	0.001	0.001	[−0.0005, 0.0015]	0.12	0.30
		Component	−0.09	0.07	[−0.23, 0.05]	−0.10	0.23	−0.10	0.11	[−0.31, 0.12]	−0.11	0.37
		Age × Component						0.002	0.01	[−0.01, 0.02]	0.03	0.76
		Age^2^ × Component						0.0001	0.001	[−0.001, 0.002]	0.02	0.88
		Δ*R*^2^									0.001	0.94
		*R*^2^				0.53	^***^				0.53	^***^
2012	Male	Age	0.02	0.004	[0.01, 0.03]	0.46	^***^	0.02	0.01	[0.01, 0.03]	0.42	^**^
		Age^2^	0.001	0.0005	[−0.0002, 0.0017]	0.17	0.11	0.0003	0.001	[−0.001, 0.002]	0.07	0.61
		Component	−0.09	0.08	[−0.25, 0.08]	−0.10	0.32	−0.17	0.13	[−0.44, 0.09]	−0.21	0.19
		Age × Component						0.004	0.01	[−0.01, 0.02]	0.06	0.67
		Age^2^ × Component						0.001	0.001	[−0.001, 0.003]	0.17	0.38
		Δ*R*^2^									0.01	0.61
		*R*^2^				0.25	^***^				0.26	^**^
	Female	Age	0.03	0.004	[0.02, 0.04]	0.67	^***^	0.03	0.01	[0.02, 0.04]	0.60	^***^
		Age^2^	−0.0004	0.0005	[−0.001, 0.001]	−0.08	0.39	−0.0004	0.001	[−0.002, 0.001]	−0.07	0.58
		Component	−0.12	0.09	[−0.30, 0.05]	−0.12	0.17	−0.11	0.13	[−0.38, 0.15]	−0.12	0.40
		Age × Component						0.01	0.01	[−0.01, 0.02]	0.10	0.46
		Age^2^ × Component						−0.0001	0.001	[−0.002, 0.002]	−0.01	0.95
		Δ*R*^2^									0.004	0.76
		*R*^2^				0.44	^***^				0.45	^***^
2013	Male	Age	0.01	0.002	[0.01, 0.02]	0.55	^***^	0.01	0.003	[0.01, 0.02]	0.49	^***^
		Age^2^	−0.00002	0.0001	[−0.0003, 0.0003]	−0.01	0.88	−0.0001	0.0002	[−0.0005, 0.0003]	−0.05	0.70
		Component	0.01	0.06	[−0.10, 0.13]	0.02	0.80	−0.01	0.08	[−0.18, 0.16]	−0.01	0.91
		Age × Component						0.003	0.004	[−0.005, 0.011]	0.08	0.47
		Age^2^ × Component						0.0001	0.0003	[−0.004, 0.001]	0.06	0.69
		Δ*R*^2^									0.004	0.72
		*R*^2^				0.30	^***^				0.31	^***^
	Female	Age	0.02	0.002	[0.02, 0.03]	0.77	^***^	0.02	0.003	[0.02, 0.03]	0.74	^***^
		Age^2^	−0.0001	0.0001	[−0.0004, 0.0001]	−0.07	0.27	−0.0001	0.0002	[−0.0005, 0.0003]	−0.04	0.68
		Component	−0.03	0.06	[−0.14, 0.08]	−0.03	0.64	0.005	0.08	[−0.16, 0.17]	0.01	0.95
		Age × Component						0.001	0.004	[−0.01, 0.01]	0.03	0.73
		Age^2^ × Component						−0.0001	0.0003	[−0.0007, 0.0004]	−0.06	0.61
		Δ*R*^2^									0.002	0.82
		*R*^2^				0.60	^***^				0.60	^***^
2017	Male	Age	0.02	0.001	[0.01, 0.02]	0.78	^***^	0.02	0.004	[0.01, 0.02]	0.79	^***^
		Age^2^	0.0002	0.0001	[0.0001, 0.0003]	0.18	^**^	0.0003	0.0002	[−0.0001, 0.0007]	0.23	0.21
		Component	−0.09	0.04	[−0.17, −0.01]	−0.12	^*^	−0.08	0.06	[−0.19, 0.04]	−0.10	0.20
		Age × Component						−0.0002	0.002	[−0.005, 0.005]	−0.02	0.93
		Age^2^ × Component						−0.00004	0.0001	[−0.0003, 0.0002]	−0.06	0.75
		Δ*R*^2^									0.0003	0.95
		*R*^2^				0.58	^***^				0.58	^***^
	Female	Age	0.02	0.001	[0.01, 0.02]	0.73	^***^	0.01	0.004	[0.01, 0.02]	0.64	^**^
		Age^2^	0.0001	0.0001	[−0.00003, 0.00024]	0.09	0.12	0.0001	0.0002	[−0.0004, 0.0005]	0.05	0.80
		Component	−0.18	0.04	[−0.26, −0.09]	−0.24	^***^	−0.19	0.06	[−0.30, −0.07]	−0.25	^**^
		Age × Component						0.001	0.003	[−0.004, 0.006]	0.09	0.63
		Age^2^ × Component						0.00003	0.0001	[−0.0002, 0.0003]	0.05	0.81
		Δ*R*^2^									0.001	0.88
		*R*^2^				0.56	^***^				0.56	^***^
2018	Male	Age	0.01	0.001	[0.01, 0.02]	0.68	^***^	0.02	0.004	[0.01, 0.03]	0.95	^***^
		Age^2^	0.0001	0.0001	[−0.0001, 0.0003]	0.07	0.31	0.00003	0.0003	[−0.001, 0.001]	0.02	0.91
		Component	−0.07	0.04	[−0.15, 0.01]	−0.11	0.10	−0.08	0.06	[−0.20, 0.04]	−0.12	0.19
		Age × Component						−0.004	0.003	[−0.009, 0.002]	−0.29	0.16
		Age^2^ × Component						0.00004	0.0002	[−0.0003, 0.0004]	0.05	0.82
		Δ*R*^2^									0.01	0.37
		*R*^2^				0.48	^***^				0.49	^***^
	Female	Age	0.02	0.001	[0.01, 0.02]	0.73	^***^	0.01	0.005	[0.005, 0.02]	0.56	^**^
		Age^2^	−0.0001	0.0001	[−0.0003, 0.0001]	−0.05	0.39	−0.0002	0.0003	[−0.0008, 0.0004]	−0.12	0.53
		Component	−0.19	0.04	[−0.27, −0.10]	−0.26	^***^	−0.20	0.06	[−0.32, −0.09]	−0.28	^***^
		Age × Component						0.003	0.003	[−0.003, 0.008]	0.17	0.36
		Age^2^ × Component						0.0001	0.0002	[−0.0003, 0.0004]	0.07	0.71
		Δ*R*^2^									0.004	0.58
		*R*^2^				0.59	^***^				0.59	^***^

*** *p < 0.001*,

** *p < 0.01*,

* *p < 0.05*.

**Figure 8:**
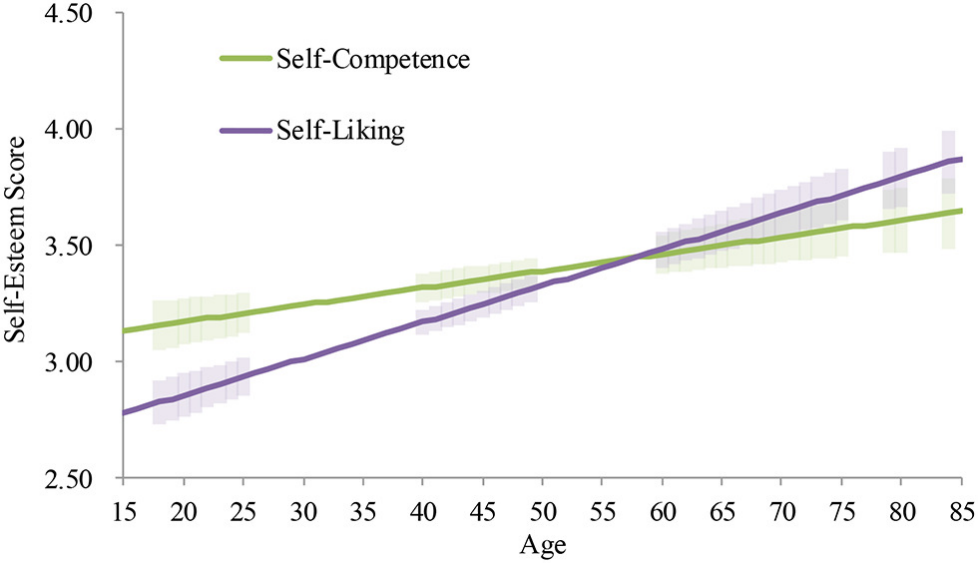
Average and predicted self-esteem scores by component across ages among females in Japan (2009 survey). Note. Error bars represent 95% confidence intervals.

For females in 2009, Step 2 significantly increased the coefficient of determination. The interaction term between age and the component significantly predicted the score. Thus, we conducted the same hierarchical multiple regression analyses on each component as we did for the global self-esteem (Table 8). Although in both components age significantly explained the scores, the slope of the increase was higher for self-liking (*B* = 0.02, *p* < 0.001) than for self-competence (*B* = 0.01, *p* < 0.001; Figure 8). Yet, both patterns consistently indicated a continuous upward trend in self-esteem.

**Table 8:** Regression models predicting each sub-component of self-esteem from age for females in the 2009 study.

		**Step 1**					**Step 2**				
**Component**		***B***	***SE***	**95% *CI***	**β**	***p***	***B***	***SE***	**95% *CI***	**β**	***p***
Self-Competence	Age	0.01	0.002	[0.004, 0.011]	0.59	^***^	0.01	0.002	[0.004, 0.011]	0.61	^***^
	Age^2^						0.0002	0.0001	[−0.0001, 0.0004]	0.21	0.13
	Δ*R*^2^									0.04	0.13
	*R*^2^				0.35	^***^				0.39	^***^
Self–Liking	Age	0.02	0.002	[0.01, 0.02]	0.87	^***^	0.02	0.002	[0.01, 0.02]	0.86	^***^
	Age^2^						−0.0001	0.0001	[−0.0003, 0.0002]	−0.06	0.52
	Δ*R*^2^									0.003	0.52
	*R*^2^				0.75	^***^				0.75	^***^

*** *p < 0.001*.

Due to the consistent relationship between self-competence and self-liking, the age patterns for each component were same as those for global self-esteem. Specifically, the quadratic increases were found among males in 2009 and both genders in 2017, and the linear increases were found in the other subgroups.

## Discussion

### Summary of the Results and Implications

We investigated age differences in self-esteem in Japan from adolescents aged 16 to the elderly aged 88 by using the RSES. Previous cross-sectional research has investigated age differences in self-esteem in Japan (12, 13, 17). However, it had two limitations: (1) it did not directly examine age differences in global self-esteem and (2) it did not investigate self-esteem among the elderly over the age of 70. These limitations had to be overcome to fully understand the developmental trajectory in self-esteem across the life span in Japan. Therefore, we examined age differences in self-esteem by conducting the RSES on more diverse sample that included the elderly over the age of 70.

First, as predicted, we found a pattern to the developmental trajectory of global self-esteem that is consistent with previous research on self-liking (12, 13, 18). We had predicted that the developmental pattern of self-esteem would be consistent with that of self-liking, but we had not had empirical evidence to support it. In this research, we empirically confirmed that self-competence and self-liking are closely associated with each other and have a consistent developmental pattern in Japan. Specifically, across the six cross-sectional surveys, the average level of self-esteem was low in adolescence, but then gradually continued to become higher from young adulthood to late adulthood. This trajectory was consistent with findings in previous research in European American cultures (2, 3).

Second, as expected, analyses showed that the average level of self-esteem continued to indicate an upward trend beyond the age of 50 in Japan. All of the six independent cross-sectional datasets consistently showed that self-esteem continued to become higher from adulthood to old age both for males and females. This finding was inconsistent with previous research that showed a drop in self-esteem over the age of 50 in European American cultures (2, 3). With old age comes a more humble, modest, and balanced perspective toward oneself, which leads to a decline in self-esteem in old age in European American cultures [e.g., Robins et al. (4); Robins and Tresniewski (3)]. Previous research has indicated that, compared to people in European American cultures, people in Japan have more humble and balanced attitudes toward themselves, not just in old age [e.g., Heine et al. (8); Heine and Hamamura (17)], which may account for the absence of a drop of self-esteem among the Japanese people over the age of 50. Thus, this research demonstrates that different developmental patterns can emerge in different social/cultural environments.

One may wonder whether the absence of a sharp drop in self-esteem in Japan is caused by the fact that participants answered the questionnaire on the internet. Elderly people who use the internet may differ from the elderly population in general (e.g., they may be wealthier and healthier). However, this was also the case in previous research that observed a clear drop in self-esteem among the elderly in the U.S. (e.g., (4)). Thus, this explanation is insufficient to account for the cultural difference in the developmental trajectory of self-esteem among the elderly. Still, it is desirable to collect more representative data especially from the elderly and see if the result is consistent with the present research.

Three datasets showed that gender differences in the pattern of age differences were absent. The other three datasets indicated that there were slight differences between gender: slopes for females were a little larger than those for males. In sum, the pattern of age differences in self-esteem was similar between gender, if any small differences. These results were consistent with previous research (2, 7, 19).

We also confirmed the measurement invariance of the Rosenberg's Self-Esteem Scale (11) across gender and age-groups in Japan. Regarding gender, five out of the six datasets demonstrated scalar and structural invariance, and one showed partial scalar and structural invariance. Thus, in all of the datasets, at least partial scalar and structural invariance were supported. Regarding age-group, one out of the six datasets showed scalar and structural invariance, four showed partial scalar and structural invariance, and one showed metric and structural invariance. Overall, in the most datasets, at least partial scalar and structural invariance were supported. These results showed that the model structure and the adequacy of the measure were invariant across gender and age-groups.

### Limitations and Future Directions

This research investigated age differences in self-esteem from adolescents in their teens to the elderly in their 80s by analyzing six cross-sectional datasets from a large and diverse sample. But, in cross-sectional data, age differences involve cohort differences. This research is a reasonable first step to understand the developmental trajectory of self-esteem across the life span in Japan. In fact, the absence of a drop in self-esteem in Japan might be caused by the cohort effect. Specifically, older cohorts might have higher self-esteem and younger cohorts might have lower self-esteem (23–26), which might obscure the drop of self-esteem in Japan. Thus, in the future, it is necessary to analyze longitudinal data which can distinguish between age differences and cohort differences.

Another limitation is that, although the sample sizes were relatively large for teens (*n* = 211), adults (*n*_20s_ = 1,161, *n*_30s_ = 970, *n*_40s_ = 1,382, *n*_50s_ = 1,056), and the elderly in their 60s (*n* = 1,031) and 70s (*n* = 253), the sample size for the elderly in their 80s was small (*n* = 49). Thus, the results for this age group may be unreliable. It would be desirable to examine this point further by collecting more representative data.

## Data Availability Statement

The datasets presented in this article are not readily available because participants were not asked to provide permission to disclose individual data at the time of data collection. Requests to access the minimal datasets (aggregate level) should be directed to Yuji Ogihara (yogihara@rs.tus.ac.jp).

## Ethics Statement

This study was carried out in accordance with the recommendations of the Declaration of Helsinki and the Japanese Psychological Association with written informed consent from all subjects. Ethical approval was not required for this study in accordance with the local legislation and institutional requirements. This study asked participants to answer one of the most frequently used questionnaires (Rosenberg Self-Esteem Scale) and this questionnaire did not include any items that may harm participants. Thus, the protocol was not submitted to an ethics committee. Both individual researchers in charge of each survey and their respective research firms confirmed that all surveys were without any ethical concerns. All subjects gave written informed consent via the online questionnaire in accordance with the Declaration of Helsinki.

## Author Contributions

YO contributed conception and design of the study, performed the statistical analysis, and wrote the first draft of the manuscript. TK collected the data and provided the critical comments on the manuscript. All authors contributed to manuscript revision and approved the final manuscript.

## Conflict of Interest

The authors declare that the research was conducted in the absence of any commercial or financial relationships that could be construed as a potential conflict of interest. The reviewer ST declared a shared affiliation, with no collaboration, with the authors to the handling editor at the time of review.

## Footnotes

^1^Bleidorn et al. (10) examined cultural variation in age and gender differences in self-esteem across 48 nations including Japan. However, with regards to cultural differences in the developmental trajectory of self-esteem, it had at least three limitations. First, it investigated age differences in self-esteem between the ages of 16 and 45, leaving it unclear how average levels of self-esteem change after early middle age. Second, it indicated that self-esteem in females showed an upward trend from early adolescence to middle adulthood in Japan whereas self-esteem in males showed a downward trend during this period. Among 48 nations, this age pattern in males was found only in Japan, but the reason for this was not discussed. Third, as mentioned as a limitation by the authors, it used only one item “I see myself as someone who has high self-esteem” (a 5-point scale ranging from 1: disagree strongly to 5: agree strongly) instead of adequately constituted scale [e.g., Rosenberg Self-Esteem Scale; Rosenberg (11)]. Although it was stated that the validity and reliability had been confirmed in the U.S. (although the item and its anchors differed from the original research), it is unclear whether the validity and reliability were confirmed in other nations including Japan. The word “self-esteem” is abstract and conceptual, so it may be interpreted differently across cultures and/or within each culture. This may have contributed to the exceptional pattern found in Japan (i.e., the continuous downward trend in males and the continuous upward trend in females).

^2^The translations differed between years is because these studies were conducted as an omnibus survey with other researchers.

^3^The other four items assessing self-competence were “I am able to do things as well as most other people.” (SC2), “I feel that I'm a person of worth, at least on an equal plane with others.” (SC3), “I feel I do not have much to be proud of.” (SC4), and “All in all, I am inclined to feel that I am a failure.” (SC5).

^4^The other four items assessing self-liking were “I take a positive attitude toward myself.” (SL2), “I certainly feel useless at times.” (SL3), “At times I think I am no good at all.” (SL4), and “I wish I could have more respect for myself.” (SL5).

^5^Although teenagers are usually not regarded as younger adults, because the size of this sample was relatively small, teenagers were conventionally included in the younger adults group in this analysis.

^6^Because the datasets in 2011 and 2012 did not include participants aged over 60 years, we split the participants into two age groups: younger adults (20s, 30s) and middle-aged adults (40s, 50s).

^7^Because the covariances between error terms of SC1, SC2, SC3, and error terms of SL1 and SL2 were large, we included them in the model (Model 1). These high associations may be due to the nature of the items: the items were affirmative, and the remaining (i.e., SC4, SC5, SL3, SL4, SL5) were reversed items. Even if we did not include these covariances in the model, the patterns of the results of multi-group CFA were consistent.

^8^We did not examine whether the developmental pattern of self-esteem (e.g., the shape of changes, the magnitude of the slope) differed among the six datasets. Naturally, the developmental pattern is different because the time periods covered in the surveys are significantly different (Tables 1, 2). Moreover, previous research has shown that the average levels of self-esteem decreased over time in Japan [(23–25); for a review of historical changes in self-esteem in Japan see Ogihara (26)]. Thus, the self-esteem score should not be simply averaged across the surveys that were conducted over nine years. Even if the self-esteem scores are z-transformed in each survey, the periods covered by the surveys are significantly different, and they should not be aggregated.

^9^In the partial scalar invariance model, item intercepts that were constrained did not include SL5 (i.e., excluding the constraint of SL5's item intercept from the full scalar model).

^10^In the partial scalar invariance models, item intercepts that were constrained were as follows. 2009: SC1, SC2, SC3, SL2; 2012: other than SL3; 2013: SC1, SC3, SL2, SL5; 2017: SC4, SL5; 2018: SC2, SL5.

^11^The reason for these differences in the levels of measurement invariance could be due to differences in factors such as total sample sizes, proportions of age-group sample size, and type of scale translations. However, we do not have sufficient data to empirically detect the reason(s). Thus, in the future, it would be important to investigate the possibility that people across a broad range of age might respond to RSES items differently.

^12^The lowest predicted score was at the age of 32 when raw data were unavailable. Thus, effect sizes were not calculated.

^13^We describe developmental patterns of self-esteem based on age differences in self-esteem. However, it should be noted that our data were cross-sectional and not cross-temporal. Age differences include not only developmental changes that an individual experiences over the life course, but also cohort differences. Thus, these age differences do not necessarily mean developmental changes. Although findings obtained from cross-sectional research on self-esteem are consistent with those obtained from cross-temporal research [for a review, see Orth and Robins (2), this should be kept in mind (also see, the Limitations and Future Directions section of the Discussion below).

^14^Effect sizes were calculated by using predicted average scores, standard deviations and sample sizes in three years (a given age, and 1 year before and after the age) to secure larger sample sizes and avoid unstable results.

^15^Comparing the slopes in linear models (Step 1) between gender, the slope for females (*B* = 0.011, *p* < 0.001) was slightly larger than that for males (*B* = 0.009, *p* < 0.001).

^16^We have also shown the results of the analysis conducted separately by gender to facilitate comparisons with other surveys (Supplementary Material; Table S1; Figure S1).

^17^We have also shown the results of the analysis conducted separately by gender to facilitate comparisons with other surveys (Supplementary Material; Table S1; Figure S2).

^18^We have also showed the results of the analysis conducted separately by gender to facilitate comparisons with other surveys (Supplementary Material; Table S1; Figure S3).
