# Supplementary material for: The Developmental Trajectory of Self-Esteem Across the Life Span in Japan: Age Differences in Scores on the Rosenberg Self-Esteem Scale From Adolescence to Old Age
Source: Front Public Health. 2020 Aug 6;8:132. doi: 10.3389/fpubh.2020.00132 (PMC7423839; doi:10.3389/fpubh.2020.00132)
Supplement: Supplementary file 1 [file Data_Sheet_1.doc]

**Supplementary Material**

**Age Differences in Global Self-Esteem by Gender (in the 2012, 2017, and 2018 Surveys)**

**2012.**

***Male.*** The model in Step 1 was significant, and the addition of the age squared term did not significantly increase the coefficient of determination (Step 2; Table S1). Thus, the linear model (Step 1) was accepted (Figure S1). Self-esteem continued to increase from the 20s to the 50s (*d* = 0.58).

***Female.*** The model in Step 1 was significant, and the addition of the age squared term did not significantly increase the coefficient of determination (Step 2; Table S1). Thus, the linear model (Step 1) was accepted (Figure S1). Self-esteem continued to increase from the 20s to the 50s (*d* = 1.10).

**2017.**

***Male.*** The model in Step 1 was significant, and the addition of the age squared term significantly increased the coefficient of determination (Step 2; Table S1). The addition of the age cubed term did not significantly increase the coefficient of determination (Step 3). Thus, the quadratic model (Step 2) was accepted (Figure S2). Self-esteem continued to increase from the teens to the 80s (*d* = 1.29).

***Female.*** The model in Step 1 was significant, and the addition of the age squared term did not significantly increase the coefficient of determination (Step 2; Table S1). Thus, the linear model (Step 1) was accepted (Figure S2). Self-esteem continued to increase from the teens to the 80s (*d* = 1.65).

**2018.**

***Male.*** The model in Step 1 was significant, and the addition of the age squared term did not significantly increase the coefficient of determination (step 2; Table S1). Thus, the linear model (Step 1) was accepted (Figure S3). Self-esteem continued to increase from the teens to the 80s (*d* = 1.02).

***Female.*** The model in Step 1 was significant, and the addition of the age squared term did not significantly increase the coefficient of determination (Step 2; Table S1). Thus, the linear model (Step 1) was accepted (Figure S3). Self-esteem continued to increase from the teens to the 80s (*d* = 1.26).

*Table S1.* Summary of regression models predicting self-esteem from age by gender (in the 2012, 2017, and 2018 surveys).

|  |  |  | Step 1 | | | | | Step 2 | | | | | Step 3 | | | | |
| --- | --- | --- | --- | --- | --- | --- | --- | --- | --- | --- | --- | --- | --- | --- | --- | --- | --- |
| Year | Gender |  | *B* | *SE* | 95% *CI* | β | *p* | *B* | *SE* | 95% *CI* | β | *p* | *B* | *SE* | 95% *CI* | β | *p* |
| 2012 | Male | Age | 0.02 | 0.01 | [0.01, 0.03] | .49 | ** | 0.02 | 0.01 | [0.01, 0.03] | .49 | ** | 0.01 | 0.01 | [-0.01, 0.04] | .37 | .32 |
|  |  | Age^2^ |  |  |  |  |  | 0.001 | 0.001 | [-0.0005, 0.0020] | .18 | .23 | 0.001 | 0.001 | [-0.001, 0.002] | .17 | .26 |
|  |  | Age^3^ |  |  |  |  |  |  |  |  |  |  | 0.00002 | 0.0001 | [-0.0001, 0.0002] | .13 | .73 |
|  |  | Δ *R*^2^ |  |  |  |  |  |  |  |  | .03 | .23 |  |  |  | .003 | .73 |
|  |  | *R*^2^ |  |  |  | .24 | ** |  |  |  | .27 | ** |  |  |  | .27 | * |
|  | Female | Age | 0.03 | 0.01 | [0.02, 0.04] | .68 | *** | 0.03 | 0.01 | [0.02, 0.04] | .69 | *** | 0.02 | 0.01 | [-0.01, 0.05] | .41 | .20 |
|  |  | Age^2^ |  |  |  |  |  | -0.0004 | 0.001 | [-0.002, 0.001] | -.08 | .53 | -0.001 | 0.001 | [-0.002, 0.001] | -.13 | .34 |
|  |  | Age^3^ |  |  |  |  |  |  |  |  |  |  | 0.0001 | 0.0001 | [-0.0001, 0.0002] | .32 | .33 |
|  |  | Δ *R*^2^ |  |  |  |  |  |  |  |  | .01 | .53 |  |  |  | .02 | .33 |
|  |  | *R*^2^ |  |  |  | .46 | *** |  |  |  | .46 | *** |  |  |  | .48 | *** |
| 2017 | Male | Age | 0.02 | 0.002 | [0.01, 0.02] | .76 | *** | 0.02 | 0.002 | [0.01, 0.02] | .81 | *** | 0.02 | 0.003 | [0.01, 0.02] | .89 | *** |
|  |  | Age^2^ |  |  |  |  |  | 0.0002 | 0.0001 | [0.00003, 0.00037] | .19 | * | 0.0002 | 0.0001 | [0.000004, 0.000364] | .17 | * |
|  |  | Age^3^ |  |  |  |  |  |  |  |  |  |  | -0.000003 | 0.000005 | [-0.00001, 0.00001] | -.10 | .58 |
|  |  | Δ *R*^2^ |  |  |  |  |  |  |  |  | .03 | * |  |  |  | .002 | .58 |
|  |  | *R*^2^ |  |  |  | .58 | *** |  |  |  | .61 | *** |  |  |  | .61 | *** |
|  | Female | Age | 0.01 | 0.002 | [0.01, 0.02] | .75 | *** | 0.02 | 0.002 | [0.01, 0.02] | .78 | *** | 0.02 | 0.003 | [0.01, 0.02] | .84 | *** |
|  |  | Age^2^ |  |  |  |  |  | 0.0001 | 0.0001 | [-0.0001, 0.0003] | .10 | .24 | 0.0001 | 0.0001 | [-0.0001, 0.0003] | .09 | .37 |
|  |  | Age^3^ |  |  |  |  |  |  |  |  |  |  | -0.000002 | 0.000005 | [-0.00001, 0.00001] | -.07 | .71 |
|  |  | Δ *R*^2^ |  |  |  |  |  |  |  |  | .01 | .24 |  |  |  | .001 | .71 |
|  |  | *R*^2^ |  |  |  | .56 | *** |  |  |  | .57 | *** |  |  |  | .57 | *** |
| 2018 | Male | Age | 0.01 | 0.002 | [0.01, 0.02] | .72 | *** | 0.01 | 0.002 | [0.01, 0.02] | .72 | *** | 0.02 | 0.004 | [0.01, 0.03] | .87 | *** |
|  |  | Age^2^ |  |  |  |  |  | 0.0001 | 0.0001 | [-0.0001, 0.0003] | .07 | .43 | 0.0001 | 0.0001 | [-0.0001, 0.0004] | .09 | .33 |
|  |  | Age^3^ |  |  |  |  |  |  |  |  |  |  | -0.00001 | 0.00001 | [-0.00002, 0.00001] | -.18 | .36 |
|  |  | Δ *R*^2^ |  |  |  |  |  |  |  |  | .005 | .43 |  |  |  | .01 | .36 |
|  |  | *R*^2^ |  |  |  | .52 | *** |  |  |  | .53 | *** |  |  |  | .53 | *** |
|  | Female | Age | 0.02 | 0.002 | [0.01, 0.02] | .78 | *** | 0.02 | 0.002 | [0.01, 0.02] | .79 | *** | 0.02 | 0.003 | [0.01, 0.03] | .95 | *** |
|  |  | Age^2^ |  |  |  |  |  | -0.0001 | 0.0001 | [-0.0003, 0.0002] | -.05 | .50 | -0.00001 | 0.0001 | [-0.0003, 0.0002] | -.01 | .92 |
|  |  | Age^3^ |  |  |  |  |  |  |  |  |  |  | -0.00001 | 0.00001 | [-0.00002, 0.00001] | -.21 | .21 |
|  |  | Δ *R*^2^ |  |  |  |  |  |  |  |  | .003 | .50 |  |  |  | .01 | .21 |
|  |  | *R*^2^ |  |  |  | .61 | *** |  |  |  | .61 | *** |  |  |  | .62 | *** |

*Note.* *** *p* < .001, ** *p* < .01, * *p* < .05.

(A) Male

(B) Female

*Figure S1.* Average and predicted self-esteem scores across ages in Japan (2012 survey)

*Note.* Error bars represent 95% confidence intervals.

(A) Male

(B) Female

*Figure S2.* Average and predicted self-esteem scores across ages in Japan (2017 survey)

*Note.* Error bars represent 95% confidence intervals.

(A) Male

(B) Female

*Figure S3.* Average and predicted self-esteem scores across ages in Japan (2018 survey)

*Note.* Error bars represent 95% confidence intervals.
